# Evaluation of efficacy and safety of DPP-4 inhibitors for Chinese elderly patients with type 2 diabetes mellitus

**DOI:** 10.1186/s13098-020-00543-1

**Published:** 2020-04-29

**Authors:** Dong-Ni Yu, Lei Qiu, Shang-Yong Ning, Li-Xin Guo

**Affiliations:** 1 Department of Endocrinology, Beijing Hospital, National Center of Gerontology, Institute of Geriatric Medicine, Chinese Academy of Medical Sciences, P. R. China, No. 1 DaHua Road, Dong Dan, Beijing, 100730 P. R. China; 2 Department of Health Care, Beijing Hospital, National Center of Gerontology, Institute of Geriatric Medicine, Chinese Academy of Medical Sciences, P. R. China, Beijing, China; 3 Department of Hematology, Beijing Hospital, National Center of Gerontology, Institute of Geriatric Medicine, Chinese Academy of Medical Sciences, P. R. China, Beijing, China

**Keywords:** Elderly patients, Type 2 diabetes mellitus, DPP-4 inhibitors, Glycosylated hemoglobin, Safety

## Abstract

**Background:**

The safety of hypoglycemic drugs should be paid more attention to in elderly patients with type 2 diabetes mellitus due to their concomitant diseases, physiological decline of liver and kidney function and cognitive decline. The aim of this study was to evaluate the efficacy and safety of DPP-4 inhibitors in elderly patients with type 2 diabetes mellitus.

**Methods:**

From January 2010 to November 2018, 300 patients with type 2 diabetes mellitus who were over 60 years old were enrolled in the outpatient clinic of Geriatric Medical Center. Their medication records and follow-up medical records were used for retrospective analysis. The duration of treatment with DPP-4 inhibitors was more than 3 months. The changes of fasting blood glucose (GLU), glycosylated hemoglobin (HbA1C), body weight, body mass index (BMI) and liver and kidney function were compared before and after treatment.

**Results:**

The average age of 300 patients (212 males and 88 females) was 73.7 ± 9.1 years old, BMI was 26.5 ± 2.8 kg/m^2^ and the duration of diabetes was 10.7 ± 8.2 years. The results of retrospective analysis showed that HbA1C decreased by 0.27% after treatment (P < 0.001). In the group of DPP-4 inhibitors used for less than 12 months, there was no difference in liver transaminase (ALT and AST) between before and after treatment, whereas in the group of DPP-4 inhibitors used formore than 12 months, liver transaminase decreased statistically compared with after treatment (P < 0.001). The incidence of fatty liver in elderly diabetic patients decreased after using DPP-4 inhibitors. There was no significant change in serum creatinine level and creatinine clearance rate in elderly patients with type 2 diabetes mellitus after treatment of DPP-4 inhibitor. In addition, the body weight and BMI of the patients decreased significantly (P < 0.001). No hypoglycemic reaction and gastrointestinal discomfort were found in the medical records.

**Conclusion:**

After DPP-4 inhibitors were used in elderly patients with type 2 diabetes mellitus, the elevated glycosylated hemoglobin could be controlled with improved safety of liver and kidney, and might have the effect of weight loss.

## Background

Diabetes mellitus is a common disease, which has a great impact on the lives of patients. It has become one of the major diseases that do great harm to human health. Moreover, diabetes is one of the common chronic diseases in the elderly patients. Data from the International Diabetes Federation (IDF) in 2017 have shown that 425 million adults worldwide suffer from diabetes, with the highest prevalence of diabetes among 65–79 years old [1]. With the development of economy, the change of life style and the aging of population, the prevalence of diabetes mellitus in the elderly patients in China has also increased significantly. Recently, the epidemiological survey in China has showed that the prevalence of diabetes mellitus in the elderly patients was 20.2%, and there were a similar number of people with impaired glucose tolerance [2]. DPP-4 inhibitor, a serine protease inhibitor on the cell surface, has the effect of inhibiting inactivation of glucagon-like peptide-1 (GLP-1) and glucose-dependent insulin secreting polypeptide (GIP), thereby increasing the level of endogenous GLP-1 and GIP, promoting insulin release, and inhibiting glucagon secretion, which increase level of insulin and play an important role in the treatment of hyperglycemia. At present, DPP-4 inhibitors have been recommended by IDF Global Guidelines for the Management of Senile Type 2 Diabetes and China Expert Consensus on Diagnosis and Treatment Measures of Senile Diabetes as first-line alternative drugs for the treatment of senile diabetes mellitus, and are widely used in elderly patients with type 2 diabetes mellitus [3].

Although the DPP-4 inhibitors currently on the market have good safety and tolerance, the decline of renal function and metabolic capacity caused by aging may lead to the difference of metabolic kinetics of drugs. Moreover, the decline of liver and kidney function in the elderly will lead to the decrease of metabolism and excretion of DPP-4 inhibitors, so it is necessary to pay more attention to the safety of DPP-4 inhibitors in elderly diabetic patients. The risk of chronic complications is higher in elderly patients with diabetes mellitus, and there are cognitive impairment, physiological deterioration of organ function, multiple drug use and solitary living problems [4]. Type 2 diabetes mellitus is a well-known risk factor for cognitive impairment, and the neuroprotective effects of DPP-4 inhibitors have been proved. Recent evidences have found that sitagliptin may be associated with improvement of cognitive function in elderly diabetic patients [5], and vildagliptin could improve the copying subdomain of cognitive function and metabolic control of the patients with type 2 diabetes mellitus [6]. However, there are still insufficient clinical studies on the efficacy and safety of DPP-4 inhibitors in elderly patients with diabetes mellitus. Elderly diabetic patients need individualized hypoglycemic regimen and evaluation of the efficacy and safety of hypoglycemic drugs. Our study retrospectively analyzed the clinical data of elderly patients with type 2 diabetes mellitus treated with DPP-4 inhibitors, in order to evaluate the hypoglycemic efficacy and safety of liver and kidney of DPP-4 inhibitors in these elderly patients.

## Methods

### Clinical data

Elderly patients with type 2 diabetes who were treated in geriatric center from January 2010 to November 2018 were considered for inclusion in this experiment. This study was approved by The Institutional Review Board of Ethics Committee of Beijing Hospital. All participants received written and oral information prior to giving written consent, and the study was performed in accordance with the Helsinki II declaration. The study was registered at Chinese Clinical Trial Registry (No. ChiCTR-OPB-17012750).

Inclusion criteria: age 60 years old and above; diagnosis of type 2 diabetes mellitus (WHO diagnostic criteria 1999); duration of treatment with DPP-4 inhibitors over 3 months; Exclusion criteria: Acute complications of diabetes including diabetic ketosis, non-ketotic hyperosmolar coma; patients with severe cardiac or respiratory insufficiency; patients with a history of cancer within 5 years; patients with abnormal thyroid function.

Finally, 300 elderly patients with type 2 diabetes mellitus were enrolled, including 212 males and 88 females, aged 60-96 years (average 73.7 ± 9.1), BMI 18.4-36.9 kg/m^2^ (average 26.5 ± 2.8), duration of diabetes 0–36 years (average 10.7 ± 8.2). Among them, 192 cases (64%) were complicated with hypertension, 196 cases (65.3%) with hyperlipidemia, 123 cases (41%) with coronary heart disease, 4 cases (1.3%) with chronic kidney disease, 2 cases (0.6%) with chronic cardiac insufficiency and 2 cases (0.6%) with peripheral vascular disease.

### Research methods

#### Study groups

According to the time of using DPP-4 inhibitor, the patients were divided into two groups: the group with medication time less than 12 months and the group with medication time more than 12 months. eGFR less than 90 mL/min/1.73 m^2^ is defined as I stage of Chronic kidney disease(CKD) [7]. Besides, according to the estimated glomerular filtration rate (eGFR), the patients were also divided into group of eGFR less than 90 mL/min/1.73 m^2^ and the group of eGFR more than 90 mL/min/1.73 m^2^.

#### Detection indicators

According to the above grouping, the outpatient records, medical records and follow-up records of all eligible patients were retrospectively analyzed. The medical history, height, weight, body mass index (BMI), medication, fasting blood glucose (GLU), glycosylated hemoglobin (HbA1C), liver function (ALT, AST and GGT), renal function (CRE, eGFR and BUN) and the incidence of fatty liver were recorded in detail. CKD-epi formula was used to calculate eGFR rate as follows:

$${\text{eGFR }} = {\text{ a }} \times \, \left( {{\text{serum creatinine}}/{\text{b}}} \right){\text{c}} \times \, \left( {0. 9 9 3} \right){\text{age}};$$

a: takes on the following values on the basis of race and gender:

Black

Women = 166

Men = 163

White/other

Women = 144

Men = 141

b: takes on the following values on the basis of gender:

Women = 0.7

Men = 0.9

c: takes on the following values on the basis of gender and creatinine measurement:

Women

Serum creatinine ≤ 0.7 mg/dL = − 0.329

Serum creatinine > 0.7 mg/dL = − 1.209

Men

Serum creatinine ≤ 0.7 mg/dL = − 0.411

Serum creatinine > 0.7 mg/dL = − 1.209

### Statistical analysis

SPSS17.0 was used for data statistical analysis. Our data were expressed as mean ± SD. Before and after treatment, paired sample T test was used for comparison between groups. The data of the incidence of fatty liver was examined by χ^2^ test. P < 0.05 was taken as statistical significant difference.

## Results

### Use of DPP-4 inhibitors and other hypoglycemic drugs

The results of medical history review showed that DPP-4 inhibitors used for patients included siglitin (100 mg, qd), saglitin (5 mg, qd), viglitin (50 mg, bid) and liglistine (5 mg, qd), and the duration of DPP-4 inhibitors ranged from 3 to 83 months with average value of 27.2 ± 18.4 months. 222 cases (74%) used DPP-4 inhibitors on the basis of the original hypoglycemic regimen, while 78 cases (26%) used DPP-4 inhibitors instead. 81 cases (27%) were treated with DPP-4 inhibitor alone. In addition, 118 cases (39.3%) were combined with metformin, 74 cases (23.7%) were combined with glycosidase inhibitors, 52 cases (17.3%) were combined with insulin, 48 cases (16%) were combined with sulfonylureas, and 23 cases (7.7%) were combined with glinides.

### Glycosylated hemoglobin decreased after DPP-4 inhibitors were used in elderly patients with type 2 diabetes mellitus

There was no significant improvement in fasting blood glucose before and after DPP-4 inhibitor administration. However, HbA1C statistically decreased by 0.27% before treatment (P < 0.001). Patients were divided into three groups according to the level of HbA1C before treatment to compare the effects of DPP-4 inhibitors at different levels of HbA1C. The results showed that the higher the HbA1C level before treatment, the lower the level of HbA1C after treatment with DPP-4 inhibitors. Especially in HbA1C > 8% group, HbA1C decreased by 1.18% (P < 0.001) (Table 1).

**Table 1:** Comparison of GLU and before and after treatment with DPP-4 inhibitors

Index	Groups	Before treatment	After treatment	The changeb(ΔA)	P
GLU (mmol/L)	1	6.79 ± 1.19	7.00 ± 1.44	0.21	0.109
	2	7.90 ± 1.54	7.97 ± 1.69	0.07	0.780
	3	9.46 ± 3.17	9.01 ± 2.51	−0.45	0.246
HbAlC (%)	1	6.49 ± 0.45	6.48 + 0.73	−0.01	0.875
	2	7.44 ± 0.25	7.39 ± 0.97	−0.05	0.618
	3	9.08±1.19	7.90 ± 1.23	−1.18	< 0.001

**Fig. 1:**
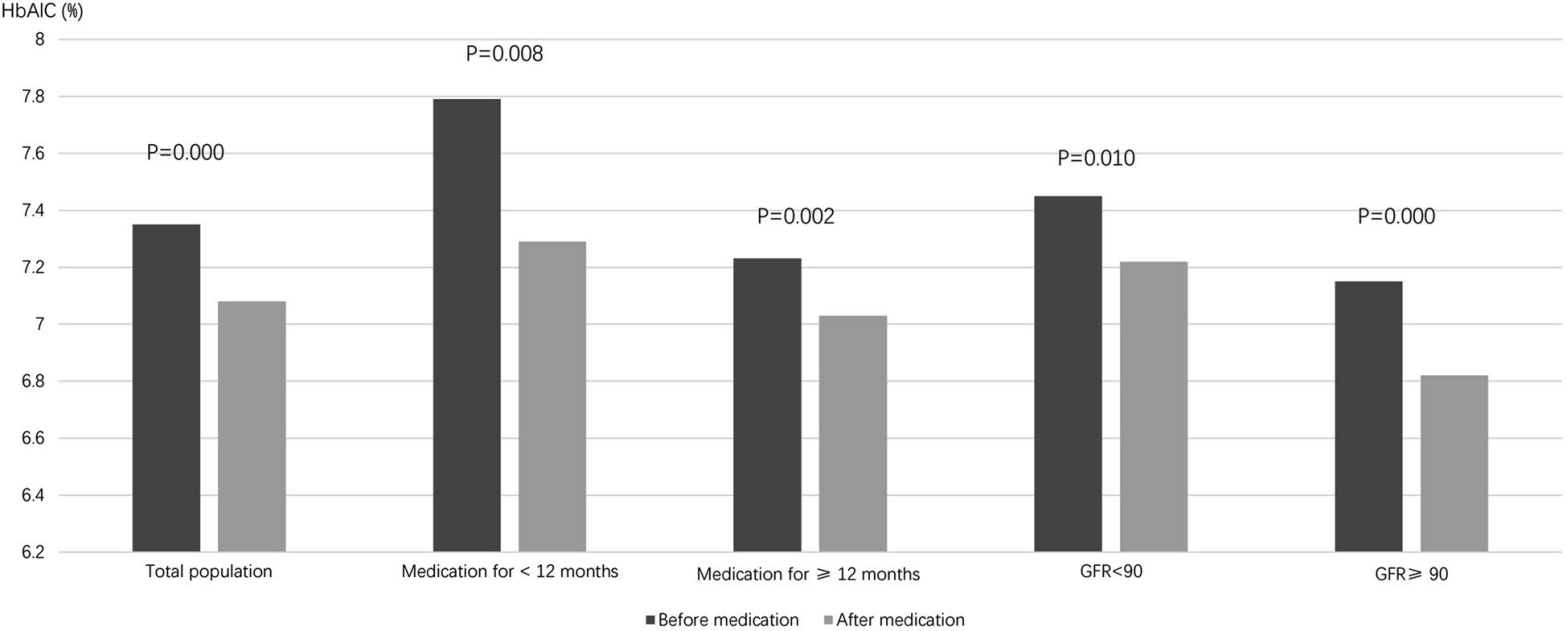
Comparison of HBA1C before and after treatment with DPP-4 inhibitor

According to the time of using DPP-4 inhibitor, the patients were divided into the group with medication time less than 12 months and more than 12 months. Besides, according to the value of eGFR, the patients were also divided into group of eGFR less than 90 mL/min/1.73 m^2^ and more than 90 mL/min/1.73 m^2^. The results of different grouping showed that HbA1C of patients was significantly lower than that before treatment (Fig. 1).

### Liver function was improved in patients treated with DPP-4 inhibitors

After DPP-4 inhibitors were used, ALT and AST statistically decreased in the group of all patients, eGFR < 90 mL/min/1.73 m^2^ and eGFR ≥ 90 mL/min/1.73 m^2^ (P < 0.001). The incidence of fatty liver in patients was also lower than that before treatment (61.5% before treatment, 55.9% after treatment, P < 0.001). It is worth noting that there was no significant difference in ALT and AST between before and after treatment in the group of DPP-4 inhibitors used for less than 12 months. However, both ALT and AST decreased in the group of DPP-4 inhibitors used for more than 12 months (P < 0.001) (Table 2).

**Table 2:** Comparison of liver function before and after treatment with DPP-4 inhibitors

Index	Groups	Before treatment	After treatment	The change (ΔA)	P
ALT	All patients	22.62 ± 10.33	20.21 ± 8.31	− 2.4	< 0.001
AST	All patients	25.83 ± 8.70	22.38 ± 10.29	− 3.4	< 0.001
GGT	All patients	29.82 ± 17.42	30.52 ± 22.55	0.7	0.567
ALT	Medication time ≤ 12	22.52 ± 8.44	22.58 ± 10.22	+ 0.07	0.955
	Medication time > 12	22.79 ± 10.84	19.75 ± 7.62	− 3.05	< 0.001
AST	Medication time ≤ 12	23.72 ± 6.55	23.85 ± 7.85	− 0.13	0.884
	Medication time > 12	26.57 ± 9.17	22.12 ± 10.94	− 4.45	< 0.001
GGT	Medication time ≤ 12	27.89 ± 13.96	28.14 ± 18.39	− 0.26	0.865
	Medication time > 12	30.19 ± 18.06	31.07 ± 23.35	+ 0.88	0.537
ALT	eGFR ≥ 90 mL/min/1.73 m^2^	23.74 ± 9.12	20.94 ± 7.78	− 2.80	< 0.001
	eGFR < 90 mL/min/1.73 m^2^	21.96 ± 10.93	19.83 ± 8.62	− 2.13	< 0.001
AST	eGFR ≥ 90 mL/min/1.73 m^2^	25.41 ± 6.76	22.70 ± 8.45	− 2.71	< 0.001
	eGFR < 90 mL/min/1.73 m^2^	26.10 ± 9.63	22.24 ± 11.22	− 3.86	< 0.001
GGT	eGFR ≥ 90 mL/min/1.73 m^2^	33.80 ± 21.78	32.64 ± 24.86	− 1.16	0.561
	eGFR < 90 mL/min/1.73 m^2^	28.13 ± 14.91	29.66 ± 21.53	1.54	0.315

### Renal function was improved in patients treated with DPP-4 inhibitors

In the groups of DPP-4 inhibitors used for less than 12 months and more than 12 months, there was no significant difference in renal function index (CRE, eGFR and BUN)between before and after treatment with DPP-4 inhibitors. In the group of eGFR ≥ 90 mL/min/1.73 m^2^, the eGFR was significantly lower than that before treatment (P < 0.001), but in the group of eGFR < 90 mL/min/1.73 m^2^, there was no significant difference in the eGFR of patients between before and after treatment (Table 3).

**Table 3:** Comparison of renal function before and after treatment with DPP-4 inhibitors

Index	Groups	Before treatment	After treatment	The change (ΔA)	P
CRE	All patients	73.80 ± 20.23	74.83 ± 20.67	1.31	0.106
eGFR	All patients	82.38 ± 5.51	81.46 ± 15.50	0.92	0.062
BUN	All patients	6.37 ± 5.97	7.94 ± 21.66	1.57	0.246
CRE	Medication time ≤ 12	62.75 ± 15.60	62.68 ± 16.45	− 0.07	0.952
	Medication time > 12	77.44 ± 20.12	78.69 ± 20.35	+ 1.25	0.105
eGFR	Medication time ≤ 12	86.31 ± 12.90	86.13 ± 13.03	− 0.19	0.845
	Medication time > 12	81.01 ± 16.02	79.98 ± 15.93	− 1.03	0.078
BUN	Medication time ≤ 12	5.57 ± 1.88	11.92 ± 45.75	6.35	0.299
	Medication time > 12	6.62 ± 6.72	6.94 ± 6.80	+ 0.32	0.628
CRE	eGFR ≥ 90 mL/min/1.73 m^2^	82.72 ± 18.85	82.58 ± 20.24	− 0.14	0.875
	eGFR < 90 mL/min/1.73 m^2^	58.42 ± 10.78	61.56 ± 12.81	+ 3.15	< 0.001
eGFR	eGFR ≥ 90 mL/min/1.73 m^2^	74.27 ± 13.27	74.10 ± 14.02	− 0.17	0.815
	eGFR < 90 mL/min/1.73 m^2^	96.92 ± 5.12	94.64 ± 6.95	− 2.28	< 0.001
BUN	eGFR ≥ 90 mL/min/1.73 m^2^	6.96 ± 7.41	9.32 ± 27.17	+ 2.36	0.273
	eGFR < 90 mL/min/1.73 m^2^	5.37 ± 1.24	5.62 ± 1.30	+ 0.24	0.079

### Weight loss occurred after patients were treated with DPP-4 inhibitors

In the group of all patients, eGFR < 90 mL/min/1.73 m^2^ and the eGFR ≥ 90 mL/min/1.73 m^2^, the weight and BMI of the patients decreased after treatment (P < 0.001), but there was no significant change in height. Besides, the there was no significant difference in weight and BMI before and after DPP-4 inhibitors were administered for less than 12 months. Nevertheless, the weight and BMI of patients who took more than 12 months of DPP-4 inhibitors statistically decreased (P < 0.001) (Table 4).

**Table 4:** Comparison of height, weight and BMI before and after treatment with DPP-4 inhibitors

Index	Groups	Before treatment	After treatment	The change (ΔA)	P
Height (cm)	All patients	166.24 ± 7.34	166.17 ± 7.34	− 0.07	0.472
Weight (kg)	All patients	73.37 ± 10.16	71.96 ± 10.00	− 1.4	< 0.001
BMI (kg/m^2^)	All patients	26.50 ± 2.80	26.00 ± 2.80	− 0.5	< 0.001
Height (cm)	Medication time ≤ 12	160.46 ± 7.69	160.45 ± 7.82	− 0.01	0.989
	Medication time > 12	167.75 ± 6.44	167.66 ± 6.43	− 0.09	0.451
Weight (kg)	Medication time ≤ 12	66.61 ± 10.41	65.98 ± 10.90	− 0.63	0.171
	Medication time > 12	75.13 ± 9.23	73.52 ± 9.06	− 1.6	< 0.001
BMI (kg/m^2^)	Medication time ≤ 12	25.87 ± 3.02	25.53 ± 3.08	− 0.34	0.065
	Medication time > 12	26.68 ± 2.69	26.13 ± 2.69	− 0.55	< 0.001
Height (cm)	eGFR ≥ 90 mL/min/1.73 m^2^	166.10 ± 7.76	166.05 ± 7.78	− 0.05	0.589
	eGFR < 90 mL/min/1.73 m^2^	166.46 ± 7.11	166.38 ± 7.11	− 0.08	0.582
Weight (kg)	eGFR ≥ 90 mL/min/1.73 m^2^	72.21 ± 11.08	70.69 ± 10.73	− 1.52	< 0.001
	eGFR < 90 mL/min/1.73 m^2^	74.00 ± 9.62	72.66 ± 9.58	− 1.34	< 0.001
BMI (kg/m^2^)	eGFR ≥ 90 mL/min/1.73 m^2^	26.06 ± 2.77	25.51 ± 2.47	− 0.55	< 0.001
	eGFR < 90 mL/min/1.73 m^2^	26.70 ± 2.78	22.22 ± 2.93	− 4.48	< 0.001

## Discussion

There are many problems in elderly diabetic patients, such as high fluctuation of blood glucose, decline of cognitive function and self-management ability, poor compliance, etc [8]. Therefore, compared with younger diabetic patients, it is more difficult for elderly patients to carry out blood glucose management. If lifestyle intervention can not effectively control blood sugar, addition of hypoglycemic drugs should be considered in time. However, in term of the selection of hypoglycemic drugs, the basic situation of elderly patients and the risk/benefit ratio should be comprehensively assessed. The characteristics of this study were that the patients in the study group were older (73.7 ± 9.1 years old), had a long course of diabetes (10.7 ± 8.2 years) and complicated with many chronic diseases. The three most common chronic diseases were hyperlipidemia (65.3%), hypertension (64%) and coronary heart disease (41%). These people are at high risk of liver and kidney side effects of hypoglycemic drugs.

The retrospective analysis showed that HbA1C decreased by 0.27% after DPP-4 inhibitor was used, and the difference was statistically significant. A randomized, controlled and placebo study of 206 type 2 diabetic patients over 65 years old with poor glycemic control was conducted. The results showed that the use of sigliptin (100 mg, *qd*, 24 weeks) reduced HbA1C by about 0.5% [9]. The overall decrease of glycosylated hemoglobin in this study is relatively small, which may be related to the long course of disease (10.79 ± 8.22 years) and the low HbA1C level (7.35 ± 1.20%) before medication. This study also found that the higher the HbA1C before treatment, the more significant the effect of DPP-4 inhibitors on reducing HbA1C. In the HbA1C > 8% group, HbA1C decreased to 1.18% since before treatment, and the difference was statistically significant. These results may be related to the glucose- dependent hypoglycemic mechanism of DPP-4 inhibitors. At the same time, compared with the patients before medication, the HbA1C decreased in both patients whose medication time was less than 12 months and more than 12 months. These results suggested that DPP-4 inhibitors could effectively control blood glucose in elderly patients for a long time. The results of this study confirmed that DPP-4 inhibitors could effectively improve HbA1C in elderly patients with type 2 diabetes mellitus.

This study also found that liver transaminases (ALT and AST) in patients with DPP-4 inhibitors for less than 12 months were not significantly different from those before treatment, while liver transaminases decreased in patients with DPP-4 inhibitors for more than 12 months. The incidence of fatty liver in elderly diabetic patients decreased after DPP-4 inhibitors were used. These results suggested that long-term use of DPP-4 inhibitors could improve fatty liver and liver function in elderly patients with type 2 diabetes mellitus. It has been found that the upregulation of DPP-4 in mouse hepatocytes could lead to insulin resistance and obvious hepatic steatosis [10]. In mice model, agritin, siglitin, tigliptin and ligliptin can alleviate hepatic steatosis, inflammation, hepatic adipogenesis and insulin resistance. It is reported that patients with type 2 diabetes mellitus complicated with nonalcoholic fatty liver disease (NFALD) were treated with aggliptin for 12 months, and the scores of nonalcoholic fatty hepatitis (NASH), ferritin, insulin and collagen IV 7S were significantly improved [11]. This improvement may be related to the effect of DPP-4 inhibitors on insulin signaling pathway in liver and the enhancement of endogenous GLP-1. However, a small sample of RCT studies found that the use of sigliptin for 12 and 24 weeks did not reduce the liver transaminase activity of patients with NFALD. DPP-4 inhibitors may be a new choice to prevent diabetic patients from progressing to NFALD, but more studies are needed to verify their effectiveness to NFALD.

Chronic kidney disease is a common complication of type 2 diabetes in the elderly. A retrospective cohort study of 12,570 elderly patients with diabetes showed that nearly half of the elderly patients with diabetes had different degrees of renal insufficiency [12]. The results of this study showed that 60.6% of the elderly patients with type 2 diabetes had renal dysfunction before treatment (eGFR < 90 mL/min/1.73 m^2^). As an important organ of the human body, kidney degenerative diseases occur with age, including glomerulosclerosis, atrophy of renal tubules, renal interstitial fibrosis, renal arteriosclerosis and renal blood flow reduction. Therefore, hypoglycemic drugs should be paid special attention to the safety of kidney. The results of this study showed that CRE and eGFR of elderly patients with type 2 diabetes mellitus did not change significantly after DPP-4 inhibitors were used. In the group of eGFR < 90 mL/min/1.73 m^2^, there was also no difference in CRE and eGFR between before and after treatment. Clinical studies have also confirmed that DPP-4 inhibitors did not affect eGFR in type 2 diabetic patients [13, 14]. Even in type 2 diabetic patients with end-stage renal disease receiving dialysis, DPP-4 inhibitors could effectively reduce blood glucose and have good renal safety [15]. DPP-4 inhibitor not only improves blood glucose, but also reduces glomerular high internal pressure through GLP-1 receptor on the kidney surface, and reduces the level of proinflammatory factors and growth factors [16, 17]. In addition, DPP-4 inhibitors are also considered to have GLP-1—independent protective effects on renal function, which can inhibit the degradation of key factors and exert natriuretic effect, inhibit inflammation, improve vascular function and cytoprotective effect in the kidney by inhibiting the degradation of key factors [18]. This effect has been confirmed mainly in preclinical models and in vitro studies, but clinical studies in type 2 diabetes mellitus are still lacking.

This study also found that there was no change in body weight and BMI before and after treatment in the group of DPP-4 inhibitors used for less than 12 months. However, in the group of DPP-4 inhibitors used for more than 12 months, the body weight decreased by 1.6 kg and BMI decreased by 0.5 kg/m^2^ compared with that before treatment, and the difference was statistically significant. These results suggested that DPP-4 inhibitors did not increase the body weight of elderly patients with type 2 diabetes mellitus, and long-term use of DPP-4 inhibitors could improve the body weight. The reasons for weight loss may be as follows: (1) food intake decreased in the elderly due to oral problems, impaired taste and olfaction, and decreased gastrointestinal function [19]; (2) 63% of the total population in this study used metformin combined with glycosidase inhibitors, and their effects of weight loss have been confirmed [20]; (3) DPP-4 inhibitors can improve the activity of endogenous GLP-1, thereby inhibiting appetite and delaying gastric emptying in elderly diabetic patients [21]. However, more clinical studies are needed to confirm the effect of DPP-4 inhibitors on weight improvement in elderly patients with diabetes mellitus. In addition, regarding other beneficial effects of DPP-4 inhibitors, our research had some other findings. By using INBODY720 machine (electrical impedance method), 39 subjects were tested for body composition and our results found that after using DPP-4 inhibitor, the values of body fat ratio (31.6% vs 30.7%, P = 0.310), waist to hip ratio (0.964 vs 0.960, P = 0.408) and visceral fat (107.6 vs 107.5, P = 0.966) had a downward trend. Due to the limited sample size and observation time, no statistical difference has been detected so far, and it will be further supplemented in the future study. These results indicated that DPP-4 inhibitors may have a certain degree of control on body fat, but further research is needed. Due to a retrospective data, the incidence of hypoglycemia and gastrointestinal reactions in elderly diabetic patients after treatment of DPP-4 inhibitors were not observed.

## Conclusion

In conclusion, long-term use of DPP-4 inhibitors in elderly diabetic patients with type 2 diabetes mellitus can effectively reduce HbA1C without weight gain, and has good safety of liver and kidney. However, it should be noted that DPP-4 inhibitors should adjust the dosage based on eGFR level in order to ensure the safety of drug use in the population with renal insufficiency. More clinical studies are expected to confirm the benefits of DPP-4 inhibitors.

## Data Availability

The datasets generated and analyzed during the current study are available from the corresponding author on reasonable request.
